# The impact of using different random-effects models in meta-analysis: a case study on the cognitive functioning of preterm-born children

**DOI:** 10.1093/aje/kwag120

**Published:** 2026-06-04

**Authors:** Kanella Panagiotopoulou, Soodabeh Behboodi, Jennifer Zeitlin, Anna Chaimani

**Affiliations:** Center of Research in Epidemiology and Statistics, Inserm, Université Paris Cité, Paris, France; Institute of Medical Biometry and Statistics, Faculty of Medicine and Medical Center, University of Freiburg, Freiburg im Breisgau, Germany; Inserm, INRAE, Centre for Research in Epidemiology and StatisticS (CRESS), Obstetrical, Perinatal and Pediatric Lifecourse Epidemiology, OPPaLE, Université Paris Cité, F-75004 Paris, France; Inserm, INRAE, Centre for Research in Epidemiology and StatisticS (CRESS), Obstetrical, Perinatal and Pediatric Lifecourse Epidemiology, OPPaLE, Université Paris Cité, F-75004 Paris, France; Oslo Center for Biostatistics and Epidemiology, Department of Biostatistics, University of Oslo, Oslo, Norway

**Keywords:** Bayesian meta-analysis, heterogeneous treatment effects, synthesis of observational data, semi-parametric models

## Abstract

Random-effects meta-analysis typically assumes a normal distribution for the underlying effects of the studies. However, under certain conditions, this assumption may not be the most appropriate. Simulation studies comparing several alternative random-effects models have found small differences in bias but larger differences in coverage probabilities and precision. To investigate the impact of using different meta-analysis models, we used a meta-analysis of 58 cohort studies comparing the cognitive functioning between preterm- and term-born children. We compared the results between 7 different random-effects models based on asymmetric distributions, mixtures of distributions, and Dirichlet process (DP) prior. Sensitivity analysis using variations of the 7 main models was also performed. While estimates for mean treatment effect were similar across models, the between-study variance estimates were identical. Asymmetric distribution models indicated studies with extreme effects and a left-shifted random-effects distribution while mixture models were less informative. DP models revealed clusters of studies with similar characteristics, suggesting that the observed heterogeneity may be partially explained by certain methodological and clinical characteristics of the studies. Overall, our study highlights that applying various meta-analysis models might not affect materially the summary estimates but may provide better insights into the underlying effects’ distribution and the explanation of between-study variance.

## Introduction

In the majority of published meta-analyses, the random-effects model is used to allow for heterogeneity across studies assuming a normal distribution for the underlying study effects.^1-3^ It has been claimed previously, though, that under certain conditions, the between-study normality assumption might not be the most appropriate.^4-6^ For instance, when studies form distinct clusters/subgroups or when studies with extreme effects are included, multimodal or skewed between-study distributions might fit better to the data.^7-12^ A common issue with published random-effects meta-analyses is also that inference usually focuses on the estimated mean effect. This is reasonable when the studies of the meta-analysis are sufficiently homogeneous and predictions based on the mean effect are clinically relevant. However, as the heterogeneity between studies increases, the risk that their mean may not reflect any of the available settings or populations also increases. In such cases, prediction intervals are usually very wide implying that drawing precise conclusions would be challenging. A recent simulation study showed that, in presence of large between-study variance, the mean of the random-effects distribution can be substantially inaccurately estimated.^13^

Although subgroup and meta-regression analyses are well-stablished methods for exploring variation of study results, random-effects models relaxing the between-study normality assumption have also been suggested in the literature.^9^^,^^14^^,^^15^ For instance, using the *t*-distribution for the random effects offers robustness against outlying studies due to its heavier tails,^9^^,^^10^^,^^15-17^ shifting the focus from merely detecting outliers to also accommodating them in the modeling apporach.^18^ Other options include the skew-normal or other asymmetric distributions.^9^^,^^10^^,^^15^^,^^16^ Mixture models that combine several parametric distributions by assigning a weight to each of them is an interesting alternative, particularly when studies involve several subpopulations or subsettings.^11^^,^^15^^,^^16^^,^^19-21^ Semi-parametric approaches, such as mixture models based on Dirichlet process (DP) priors,^22-24^ offer further flexibility as they can suspect potential clustering of the underlying effects without necessarily requiring data on the characteristics that cause the between-study variability.^14^^,^^25-28^ While these alternative models provide increased flexibility and may capture better the shape of the data, they often require a larger sample size and are computationally intensive.^13^^,^^26^

Previous simulation studies that compared several alternative random-effects meta-analysis models with the normal model found small differences in bias but larger differences in coverage probability and precision.^10^^,^^13^^,^^16^^,^^25^^,^^26^ They also suggested that models based on skewed extensions of the normal or the *t*-distribution have better fit to meta-analytic datasets involving outlying studies^29^^,^^30^ and mixture models when effect modifiers operate.^31^ On the other hand, it is still doubtful whether increasing model complexity leads to more accurate effect estimates compared to the widely used normal random-effects model. In this article, we aim to explore, in practice, the similarities and the discrepancies among several meta-analysis models that make different between-study assumptions in terms of summary estimates, between-study variance, predictions, and model fit. To this end, we use a meta-analysis of cohort studies comparing the cognitive functioning between preterm- and term-born children and we apply several random-effect models previously identified through a systematic review.^13^

## Methods

### Data source: meta-analysis of the cognition of children born very preterm

Preterm-born children are at an elevated risk of developmental challenges and disorders.^32^ We used a meta-analysis of 58 cohort studies investigating intelligence quotient (IQ) differences between children born extremely (EPT) or very preterm (VPT) (ie, less than 28 or 32 weeks of gestation, respectively) and those born at term (37 weeks or more).^33^ We excluded 3 studies^34-36^ from the original dataset of 61 studies that reported multiple estimates from gestational age subgroups. Standardized mean differences (SMDs) of the children’s IQ scores measured between the ages of 4 and 7 years old were used as effect measure, to account for the different measurement scales (ie, different tests) of IQ across studies and ensure comparability on a common scale. The IQ score was lower for preterm children compared to those born at term with a summary SMD of –0.87 (–0.98, –0.77). Considerable heterogeneity ${\tau}^2=0.11$ was also observed among studies (Figure 1). Following Behboodi et al.,^33^ we explored possible sources of heterogeneity by performing univariable and multivariable linear and nonlinear meta-regressions using as covariates the following clinical factors: median birth year, mean gestational age, type of IQ test^37^ (full-scale vs short-form), consideration of socioeconomic factors (use of a matched design or statistical adjustments), and study quality (based on Newcastle–Ottawa scale^38^). We also performed subgroup analyses based on level of prematurity (EPT only vs VPT) and conducted meta-regressions within each subgroup using median birth year as covariate. However, these analyses failed to explain sufficiently the observed heterogeneity (Table S1). While the spline-based meta-regressions suggested a nonlinear pattern in IQ differences across median birth years, this association was not statistically significant (*P* = 0.14, 2-sided) and did not reduce the between-study heterogeneity. These findings imply the existence of a mechanism that differentiates the studies, which requires further investigation.

**Figure 1:**
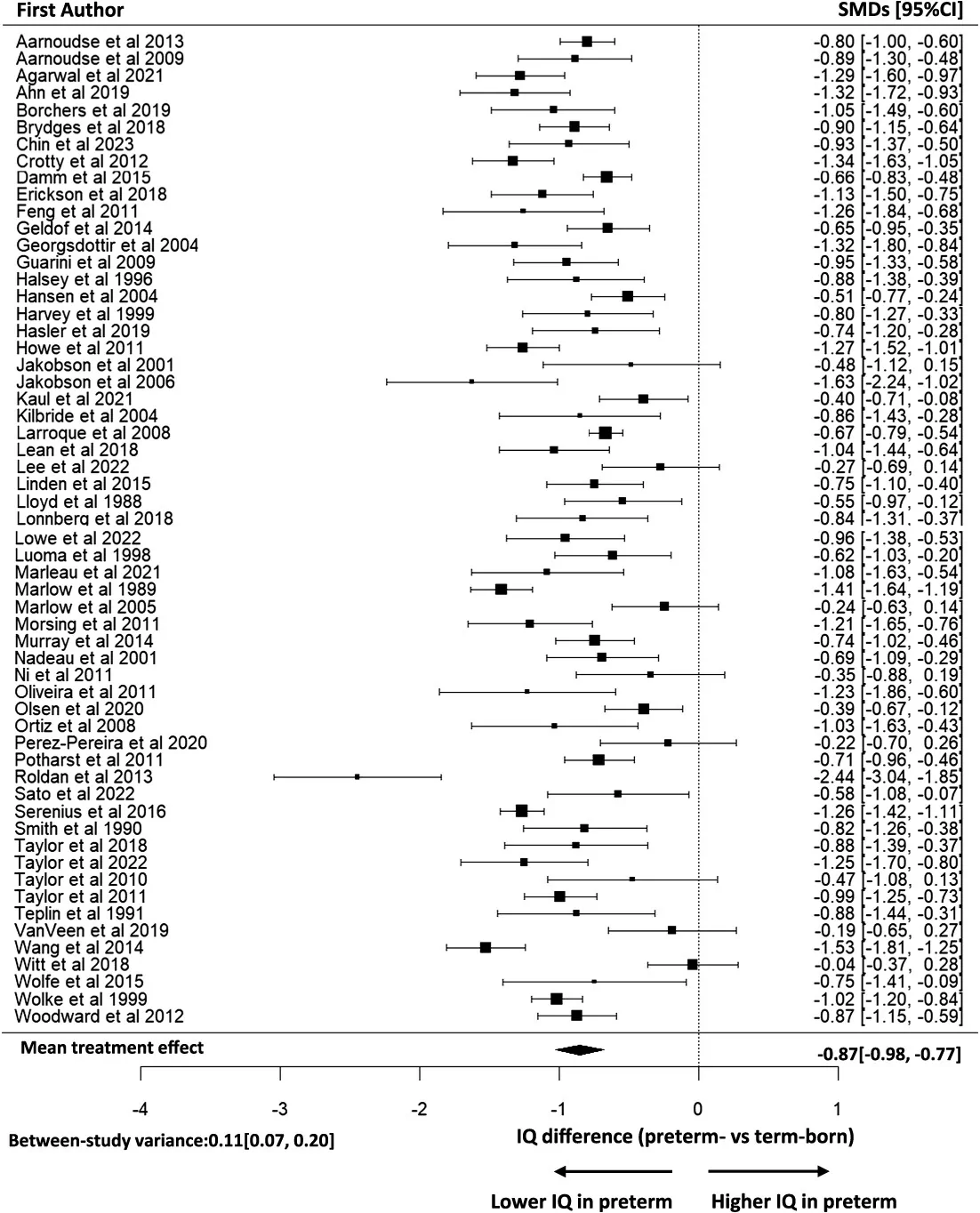
Forest plot of the 58 studies evaluating the intelligence quotient (IQ) differences between preterm- and term-born children. The between-study variance was estimated using the restricted maximum likelihood (REML) estimator. Abbreviation: SMDs, standardized mean differences.

### Alternative meta-analysis models

We first fitted a Bayesian normal random-effects model and assessed its adequacy using posterior predictive checks^39^ (Figure S1). These diagnostics indicated departures from the normality assumption, such as a higher frequency of studies with extreme effects than expected under the normal distribution, motivating consideration of more flexible random-effects distributions, including asymmetric and parsimonious mixture models. Consequently, we applied 7 different models implemented in the Bayesian framework. For all models, we used a normal approximation of the within-study distribution but employed different between-study distributions:

#### Normal distribution

This is the commonly applied random-effects model where, for every study *i=1,…, n* in the meta-analysis, we assume that


\begin{equation*} {Y}_i\sim N\left({\theta}_i,{\sigma}_i^2\right) \end{equation*}

and


(1) \begin{equation*} {\theta}_i\sim N\left(\mu, {\tau}^2\right) \end{equation*}

with ${Y}_i$, ${\theta}_i$ the observed and underlying study-specific relative effects, respectively, ${\sigma}_i^2$ the variance of ${Y}_i$ (usually treated as known), and *μ* and ${\tau}^2$ the mean and variance, respectively, of the random-effects distribution. These are also known as summary treatment effect and heterogeneity. All subsequent models focus on alternative options for equation (1).

#### T-distribution

Here, the between-study distribution is defined by the location (*ξ *), scale (*ω *), and degrees of freedom (*ν*) parameters. The location and scale parameters determine the mean (*μ*) and the variance (${\tau}^2$) of the random effects, while the degrees of freedom parameter determines the frequency of extreme effects in the data. We used vague priors for the location and scale parameters while *ν* was assigned a prior restricted to values greater than 2^9^ to ensure that *μ* and ${\tau}^2$ are well defined. Values of *ν* close to 2 indicate that studies with extreme effects occur more often, while higher values make the distribution resemble the normal. This flexibility helps mitigate the influence of studies with unusually large or small effects, reducing their impact on the overall results.^9^^,^^10^^,^^16^

#### Skew-normal and skew-t distributions

This is an extension of the normal model, which is particularly useful when studies with divergent results tend to be concentrated on one side of the distribution. In addition to the location and scale parameters, this distribution accommodates asymmetry by including an extra shape parameter (*a*) that regulates both skewness and excess kurtosis.^9^^,^^10^^,^^40^ The former determines the direction of the distribution since positive or negative skewness shifts the distribution to the right or left; 0 skewness corresponds to the normal distribution. Excess kurtosis measures how often extreme values occur compared to a normal distribution. A vague prior was assigned to *a*. The skew-*t* distribution extends the *t*-distribution by incorporating an additional shape parameter (*a*) which controls skewness similarly to the skew-normal model and reduces to the *t*-distribution when *a* = 0.^9^^,^^10^^,^^41^ In that case, the shape parameter was defined using the same prior, as for the skew normal case. In the skew-*t* distribution, the degrees of freedom parameter influences excess kurtosis. As *ν* increases, excess kurtosis approaches 0, indicating the absence of studies with extreme effect sizes. When both, skewness and excess kurtosis are 0, the distribution reduces to normal. We constrained the prior used for *ν* to values greater than 4 to ensure that both skewness and excess kurtosis are well defined and interpretable.^42^

#### Mixture of 2 normal or 2 t-distributions with different means and variances

When the data seem to naturally come from 2 or more subpopulations, a finite mixture of distributions can be considered. Here, the number of component distributions must be specified in advance. Then, studies are assigned to components based on unique probabilities (ie, weights) which are either defined a priori or incorporated into the model. The choice of component distributions can vary. The mean and variance of each component may be allowed to vary independently or remain common across components. We considered a mixture of 2 normal and a mixture of 2 *t*-distributions with component-specific means and variances defined by vague prior distributions. To specify the component weights, we used a noninformative prior to avoid imposing strong assumptions on the resulting mixture distribution. Additionally, for the mixture of 2 *t*-distributions model, we set a prior to *ν* with *ν >2*, allowing the model to more flexibly capture studies with extreme effects.

#### Mixture of point masses based on DP priors

A more flexible option to model the random-effects distribution is through a class of semi-parametric prior distributions, namely, DP priors.^24-27^^,^^43^ Such priors offer the opportunity to automatically identify clusters of studies with similar effects. Hence, they are particularly interesting when studies seem to form subgroups which often happens when effect modifiers operate across studies.

The DP priors include 2 main parameters: the base distribution and the concentration parameter. The base distribution (${F}_0$) represents our best “guess” of the random-effects distribution, while the concentration parameter ($\alpha \ge 0$) indicates how strongly the distribution of the true effects is expected to resemble the base distribution.^26^ Higher values of the concentration parameter suggest that the prior used is “closer” to the base distribution. The base distribution can also be selected through a variety of parametric distributions. The DP prior was specified as a mixture of point masses yielding a discrete distribution for the random effects where its mean was defined as a weighted average of the point masses.^26^ Study clustering is data-driven and examined by the probability that pairs of studies share a common underlying effect, known as co-clustering probability.^44^

The DP was implemented assuming a normal base distribution and employing the stick-breaking modeling approach,^45^ which further required the specification of a truncation point to ensure a reasonable approximation to the full process.^45^ The truncation point was set equal to 26, linked to the prior distribution used for the concentration parameter.^26^

Table S2 summarizes the prior specifications of the Bayesian models applied in the main analysis. All models were compared using commonly reported estimates in the literature^46-48^ (Table S3).

### Sensitivity analysis

Sensitivity analysis included several variations of the main analysis models. In the Frequentist framework, we implemented both normal and *t*-distributions,^10^^,^^16^ with the latter incorporating a test for detecting studies with extreme effects while accounting for them in the estimation via downweighing.^49^ We also applied mixture models, including a mixture of 2 normal distributions^15^ with a common component mean but different variances: a larger one capturing extreme effects and a smaller accommodating relatively homogeneous studies. The mixing probability was estimated from the data to reflect the overall proportion of studies expected to have “unusual” effects. We further implemented an extension of the above mentioned model,^50^ in which the probability that a study exhibits an extreme effect is modeled as a function of its additional variance, beyond that of the homogeneous studies. In addition, we applied a skewed model^50^ comprising a mixture of a normal distribution for homogeneous studies and an exponentially modified normal for extreme studies. Both components share a common mean but have different variances, where the mixing probabilities are defined similarly to the previous model. We repeated the primary analysis using a skeptical prior on the model-specific parameters related to the pooled SMD, which assigns low prior probability to implausibly large effects.^51^ Within the Bayesian framework, another *t*-distribution model, constructed as a mixture of scale normal distributions, was applied, with scale weights containing information about the underlying studies, thereby reducing the influence of studies with conflicting evidence.^52^ We also assessed how the base distribution, the concentration parameter, and the truncation point may influence the performance of the DP model. Specifically, we considered a skew-normal and a skew-*t* as base distributions and we examined different prior specifications for the concentration parameter combined with several truncation points.^25^^,^^26^ Finally, we included the 3 studies^34-36^ that were excluded from our primary analysis, using the DP model to assess their cluster allocation. Table S4 presents the prior distributions and definitions of model parameters for all models included in the sensitivity analysis.

### Implementation

All Bayesian models were implemented in JAGS^53^ and STAN^54^ through the “R2jags,”^55^ “rjags,”^56^ “neojags,”^57^ and “cmdstanr”^58^ packages in R. The convergence of the models was checked with traceplots and by extracting the ratio of between-chain to within-chain variance $\hat{R}$ with $\hat{R}<1.05$ suggesting convergence. For the Frequentist models, we used the “metafor”^59^ and “metaplus”^49^ R packages, as well as R codes provided by the original authors.^50^ The data and all R codes for the applied models are available on GitHub.^60^

## Results

### Summary estimates

Table 1 summarizes the estimated differences in IQ between preterm- and term-born children across the 7 applied models. All alternative models consistently resulted in lower IQ scores for preterm children with very small differences in the corresponding Credible Intervals (CrI) and were very close to the results of the (Bayesian) normal model with an SMD of –0.89 (–0.99, –0.77). Importantly, all models estimated a 100% posterior probability that preterm-born children have lower IQ scores than term-born children (Table 1). With respect to between-study variance, all models yielded consistent estimates (Table 1).

**Table 1:** Summary estimates of the mean IQ differences between preterm- and term-born children for all 7 models, together with the between-study variance and their CrI and PrI intervals, as well as the probabilities that the mean IQ of preterm children is lower than that of term-born children and the probabilities that a new study will find lower IQ scores in preterm- compared to term-born children.

**Random-effects distribution**	**SMD with 95% CrI**	**Between-study variance with 95% CrI**	**95% PrI**	$\boldsymbol{\Pr}\left(\mathbf{SMD}<\mathbf{0}\right)$	$\boldsymbol{\Pr}\left({\mathbf{SMD}}_{\mathbf{new}}<\mathbf{0}\right)$
**Normal**	–0.89 (–0.99, –0.77)	0.14 (0.08, 0.22)	(–1.65, –0.15)	1	0.99
***t*-Distribution**	–0.87 (–0.96, –0.79)	0.14 (0.09, 0.24)	(–1.48, –0.27)	1	0.98
**Skew-normal**	–0.89 (–0.99, –0.80)	0.14 (0.09, 0.21)	(–1.58, –0.36)	1	1
**Skew-*t***	–0.89 (–0.99, –0.80)	0.14 (0.09, 0.23)	(–1.58, –0.37)	1	1
**Mixture of 2 normal distributions**	–0.89 (–1.00, –0.78)	0.14 (0.08, 0.25)	(–1.71, –0.17)	1	0.99
**Mixture of 2 *t*-distributions**	–0.88 (–0.98, –0.77)	0.14 (0.08, 0.27)	(–1.62, –0.10)	1	0.99
**DP**	–0.90 (–1.01, –0.79)	0.14 (0.08, 0.28)	(–1.62, –0.22)	1	1

Abbreviations: CrI, corresponding credible intervals; DP, Dirichlet process; IQ, intelligence quotient; PrI, prediction interval; SMD, standardized mean difference.

We also estimated the predicted IQ differences for a new study using the above models. The models with *t*- and skewed random-effects distributions provided the narrowest prediction intervals. Additionally, the probability that a future study would observe lower IQ scores among preterm-born children was close to 1 for all models (Table 1).

### Model-specific results

The relatively low degrees of freedom parameters estimated by the *t*-distribution (*ν =7.77*) and the skew-*t* (*ν =11.08*) models suggest the presence of studies with potentially extreme effects. In the skew-normal model, skewness and excess kurtosis parameters are theoretically bounded approximately by [–1, 1]^42^ and [0, 0.87],^42^ respectively. Here, the estimated skewness of –0.71 [–0.94, 0] near the lower bound, combined with high excess kurtosis of 0.55 [0.01, 0.81], indicates more extreme effect sizes on the left. The skew-*t* model (restricted to *ν* > 4) allows wider range for skewness [–4 to 4]^42^ and a positively unbounded excess kurtosis as *ν* approaches the value of 4. This model also suggests slightly left-shifted distribution and unbalanced effect sizes with skewness of –0.96 [–1.91, 0.17] and excess kurtosis of 1.94 [0.51, 12.14]. Figure 2A illustrates how these models may capture different patterns in the study effects in comparison with the normal model while requiring only 1 or 2 additional parameters.

**Figure 2:**
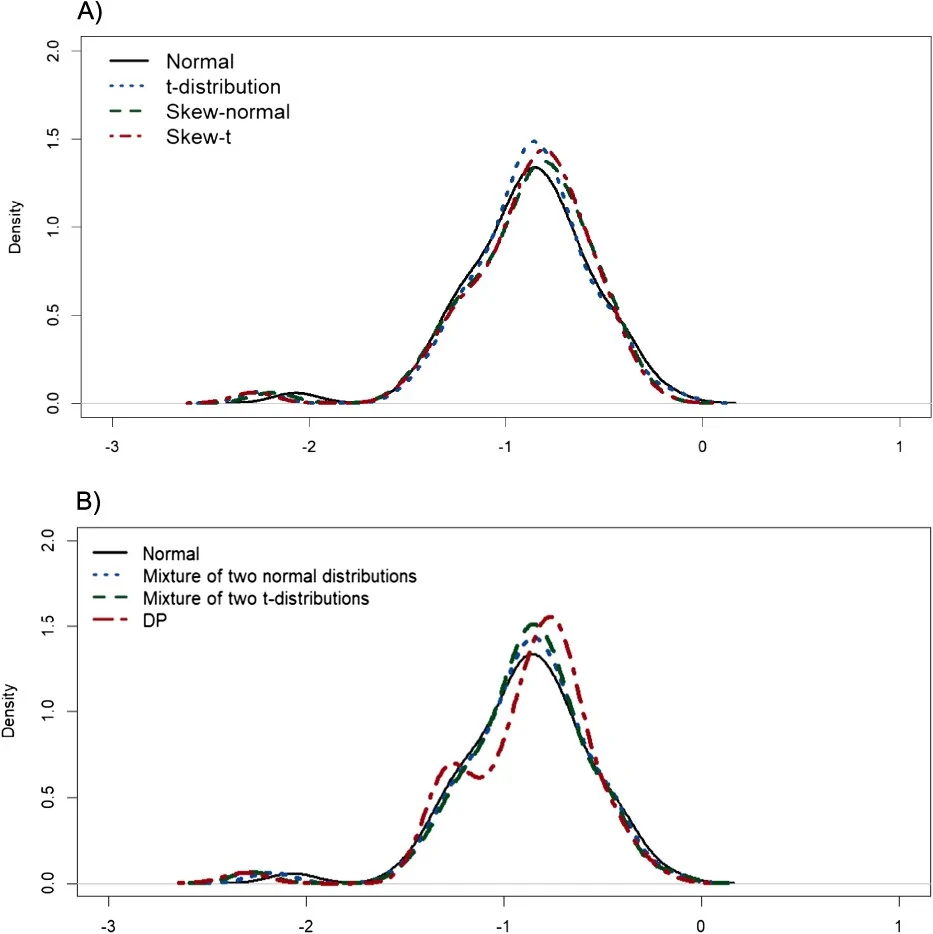
Density plots of the estimated study-specific effect sizes using (A) the normal, *t*-distribution, skew-normal, and skew-*t* models, and (B) the normal, mixture of 2 normal distributions, mixture of 2 *t*-distributions, and Dirichlet process models.

The mixture models combining 2 normal or 2 *t*-distributions, produced very similar estimates for the underlying study-specific effects (Figure 2B). Figure S2 highlights studies with different patterns in both models. In the mixture of 2 normal distributions model, only Roldan et al.’s^61^ and Wang et al.’s^62^ studies were assigned to a different component. In contrast, the mixture of 2 *t*-distributions model assigned studies to both overlapping components, with several studies showing component membership probabilities close to 0.50, indicating greater allocation uncertainty. The first component showed higher ratio of EPT to VPT studies than the second component and had mean gestational age below 28 weeks. Notably, most studies assigned to the first component were also included in cluster 3 of the DP model (see below). Component means and variances were broadly consistent between models, although component weights were more similar in the mixture of 2 *t*-distributions model, suggesting weak separation between its components (Table S5). Overall, posterior diagnostic plots indicate^63^ that the observed between-study heterogeneity may not correspond to distinct subgroups, as it appears to be driven primarily by a small number of more extreme studies, with little evidence supporting the need for a second mixture component (Figure S3). This implies that mixture models may be more informative when the data structure indicates clearer subgroup separation.

The model using a DP prior for the random effects generated a quite distinct posterior distribution for the underlying study effects in comparison with that from the normal model (Figure 2B). The heatmap of co-clustering probabilities^64^ (Figure 3) shows substantial uncertainty in classification, with many study pairs exhibiting only low to moderate probabilities of being grouped together. While the overall pattern suggests 2 broad clusters, these are not well separated, and several studies are not consistently grouped with others (eg, Roldan et al.^61^). Clustering based on the highest co-clustering probabilities between pairs of studies suggested that studies can be grouped into 2–3 main clusters, alongside multiple smaller clusters of 2–4 studies (Figure S4). Roldan et al.’s^61^ study was assigned to cluster 3 but with near-zero probability of co-clustering suggesting that it is isolated. We further investigated the potential association between the resulting clusters and available study-level characteristics. We found that the key difference between them may lie in the composition of EPTs and VPTs and their gestational ages. Clusters 1 and 3 both include high proportion of VPTs but differ in mean gestational age across their studies, with mean gestational age above 28 weeks in cluster 1 and below 28 weeks in cluster 3. Additionally, cluster 3 mostly includes unmatched/unadjusted studies that used short form of IQ test. Cluster 2 is characterized by the highest EPT to VPT ratio and a mean gestational age below 28 weeks, which may somewhat explain its tendency to overlap with cluster 3 (Figure 3). Overall, these patterns indicate that heterogeneity may be partially explained by the available characteristics, but additional factors need to be considered. While several other characteristics were examined, only these showed consistent associations with the observed clusters. Hence, in mixture models, attention should also be given to the cluster/component specific results, which might be more informative than the overall mean effect. Finally, the estimated concentration parameter is relatively high (3.57 [1.20, 4.93]), suggesting that the underlying distribution is not too different from the normal (ie, the base distribution).

**Figure 3:**
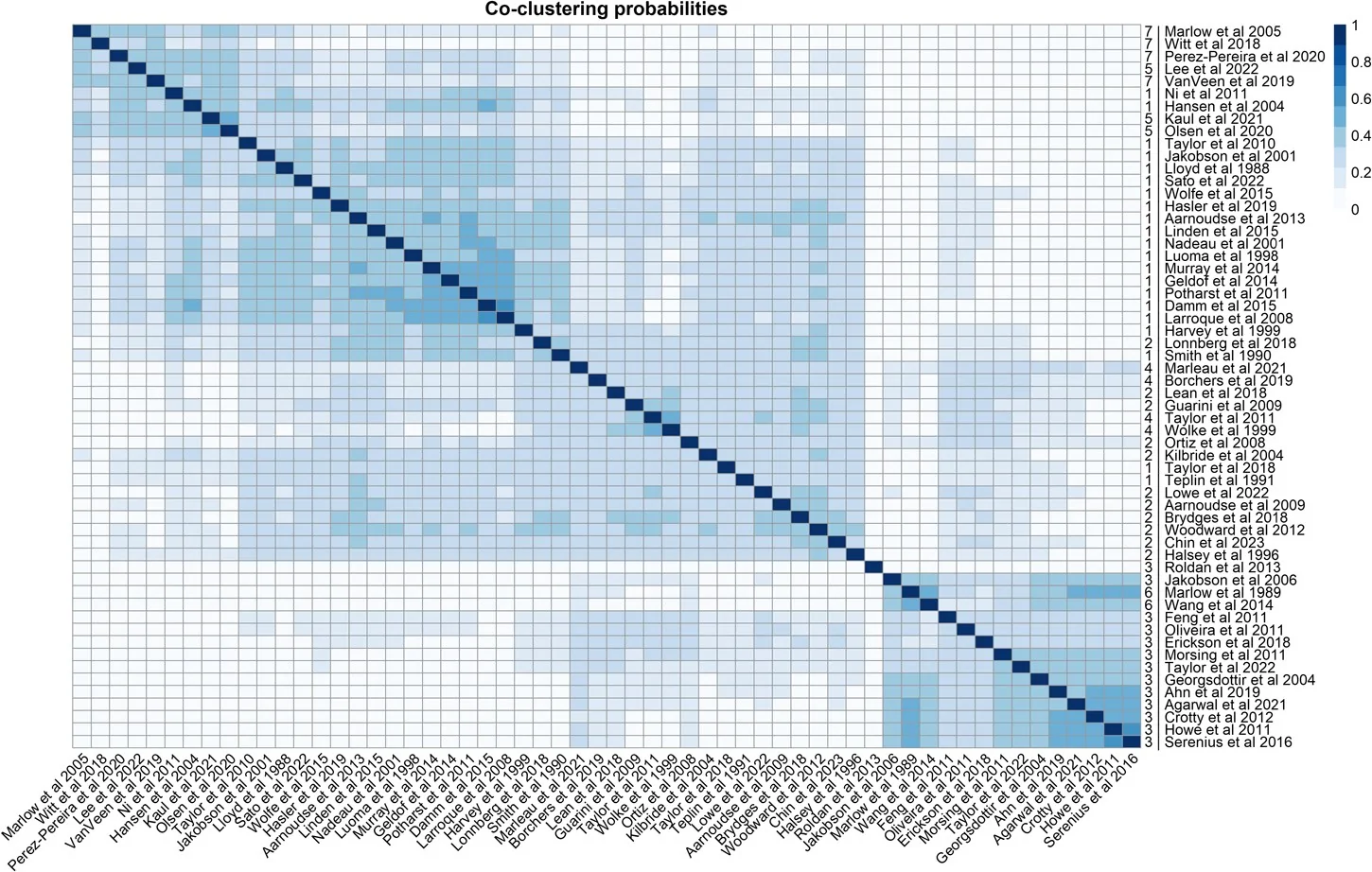
Heatmap of pairwise co-clustering probabilities between studies under the Dirichlet process model. Columns correspond to first author names of the studies and rows correspond to cluster membership for each study based on the highest co-clustering probability and the first author names. Darker blue colors indicate a higher probability that 2 studies are grouped together.

### Sensitivity analysis

Sensitivity analysis yielded similar results in terms of the overall estimates (Table S6). The Frequentist *t*-model^10^^,^^16^ did not detect studies with extreme effects (*P* = 0.11, 2-sided) although its degrees of freedom estimate (around 7) indicates the presence of studies with “unusual” effects. On the contrary, the mixture of normal and exponentially modified normal distributions model confirmed the presence of studies with extreme effects, producing an asymmetric distribution to the left (skewness*≈*0.75 and excess kurtosis*≈*1.25). The mixture of 2 normal distributions with common component mean models suggested the presence of studies with extreme effects. They identified 2 studies with divergent results (Roldan et al.^61^ and Witt et al.^65^ with probabilities 85% and 18%, respectively) and indicated that extreme studies contributed an additional variance of 0.13 (Table S6). Under the skeptical prior, the magnitude of the effect and the between-study heterogeneity remained consistent with the primary analysis, confirming lower IQ in preterm- compared with term-born children. The mixture of scale normal distributions model identified 10 studies, including Roldan et al.,^61^ as having large scale weights (Figure S5), indicating reduced consistency with the overall evidence. These studies contributed less to the estimation, yielding slightly lower SMD and heterogeneity estimates (Table S6). Using more flexible base distributions with the DP model, produced summary results and overall classification of studies into potential clusters similar to the original DP model. Incorporating skew base distributions increased the likelihood of studies with less extreme effects being grouped with more homogeneous studies, resulting in cluster overlap. Increasing the truncation point from 26 to 51 with a wider prior for the concentration parameter^26^ produced similar clustering to the original DP model but with larger uncertainty while setting it equal to the number of studies and assuming a very narrow prior for *α*^66^ ($\varGamma \left(1,1\right)$) led to more distinct clustering structures. Using the DP model, the 3 additional studies^34-36^ were assigned to clusters 1 and 2, probably because they include more VPTs than EPTs and relatively large gestational ages.

## Discussion

In the present article, we applied several meta-analysis models that relax the between-study normality assumption using data from a meta-analysis of cohort studies comparing the IQ between preterm- and term-born children. Our aim was to investigate the structure of the data and get insights about the unexplained heterogeneity. We considered models that assume non-normal random-effects distributions, including *t*-distributions, skewed distributions, mixtures of parametric distributions, and semi-parametric approaches, such as mixtures based on DP priors. We compared them with the normal model and between them in terms of the summary results and their ability to explain residual heterogeneity.

We observed negligible differences in the estimation of the mean treatment effect across all models and identical between-study variance estimates. The skewed and *t*-distribution models produced an asymmetric distribution of the study effects, allowing for higher chance of occurring studies with divergent results, something that cannot be adequately captured by the normal distribution. The mixture of 2 normal distributions model assigned most studies to a single component, whereas the mixture of 2 *t*-distributions model yielded overlap between the 2 components. The semi-parametric model based on a DP mixture of points prior revealed the presence of 2–3 main clusters of studies, as well as few studies with potentially extreme effects. The resulting clustering of studies together with the co-clustering probabilities suggested that heterogeneity may be partially explained by differences in the mean gestational age, the level of birth prematurity, type of IQ test, and the use of matched/adjusted or unmatched/unadjusted data. Examining whether the proportion of EPT children within the VPT subgroup contributes to the observed clustering could be of interest, although such data are rarely available. Although publication year did not appear to explain the formation of the clusters (Figure S4), restricting the analysis to studies published within the last 10 years resulted in a slight reduction in the IQ difference between preterm and term-born children (Figure S6). Additionally, the estimated concentration parameter gave insight regarding the shape of the true distribution, guiding model selection. Sensitivity analysis using variations of the 7 applied models under both, Bayesian and Frequentist frameworks did not change the overall conclusions.

The main limitation of this study is that the dataset does not appear to contain distinct subgroups, which restricts our ability to fully assess the performance of the mixture models. While the large number of studies allowed us to compare various meta-analysis models, our results are not generalizable to meta-analyses with fewer studies. In addition, we compared the results of the different models considering a difference of 0.2 between two SMDs as an important difference.^67^ However, using a predefined threshold for clinical importance, informed by expert opinion, would be preferable. Another challenge is the inclusion of studies that reported matched/adjusted effect sizes with those that reported unmatched/unadjusted effect sizes across various socioeconomic factors, which may inflate the heterogeneity between studies and complicate the differentiation of study subgroups with similar characteristics.

In this dataset, the different random-effects models did not materially affect the summary estimates. Nevertheless, such models may still assist to investigate residual heterogeneity, that cannot be explained by the available clinical factors, and provide better insights into the distribution of the underlying effects (Figure 2). In particular, employing asymmetric distributions for the random effects offer insights into data structure, while semi-parametric models such as the DP can assist to reveal the impact of certain characteristics and their combination by automatically identifying homogeneous subgroups of studies. In this context, although the mean treatment effect is the primary parameter of interest, greater emphasis should also be placed on additional model-specific parameters, which provide information beyond the overall mean. Supplementary summary statistics, such as prediction intervals and other relevant probabilities, can also help to meaningfully interpret the meta-analytic findings.

## Supplementary Material

Web_Material_kwag120

## Data Availability

The data underlying this article are available in GitHub, at Kanella-web/R-codes-Non-normal-meta-analysis-models-case-study.
