# Supplementary material for: The impact of using different random-effects models in meta-analysis: a case study on the cognitive functioning of preterm-born children
Source: Am J Epidemiol. 2026 Jun 4;195(11):2972–80. doi: 10.1093/aje/kwag120 (PMC13733527; doi:10.1093/aje/kwag120)
Supplement: Supplementary file 1 [file web_material_kwag120.zip › Suppl_material_The_impact_of_using_different_random_effects_models_in_meta-analysis.docx]

**Supplementary material**

**Article title**

The impact of using different random effects models in meta-analysis: A case study on the cognitive functioning of preterm-born children

**Byline**

Kanella Panagiotopoulou^1,2^, Soodabeh Behboodi^3^, Jennifer Zeitlin^3^, Anna Chaimani^4^

^1^Institute of Medical Biometry and Statistics, Faculty of Medicine and Medical Center,-University of Freiburg, Freiburg im Breisgau, Germany

^2^Université Paris Cité, Center of Research in Epidemiology and Statistics, Inserm, Paris, France

^3^Université Paris Cité, Inserm, INRAE, Centre for Research in Epidemiology and StatisticS (CRESS), Obstetrical, Perinatal and Pediatric Lifecourse Epidemiology, OPPaLE, F-75004 Paris, France

^4^Oslo Center for Biostatistics and Epidemiology, Department of Biostatistics, University of Oslo, Oslo, Norway

**Included Materials**

**Supplementary Tables**

**Table S1.** Univariable and multivariable meta-regression results.

**Table S2.** Prior distributions used for the parameters of the Bayesian models of the main analysis.

**Table S3**: Mean and variance of the different random-effects distributions.

**Table S4.** Prior distributions and definition of model parameters for all the models used in the sensitivity analyses.

**Table S5.** Estimated mean, variance and weight for each component from models using mixtures of two normal and two t-distributions.

**Table S6.** Summary estimates of the mean Intelligence Quotient (IQ) differences between preterm- and term-born children for all models used in the sensitivity analysis, together with the between-study variance and their corresponding confidence (CI) or credible (CrI) and prediction (PrI) intervals. For Bayesian models, the probabilities that the mean IQ of preterm children is lower than that of term-born children and the probabilities that a new study would find lower IQ scores in preterm compared with term-born children are also presented.

**Supplementary Figures**

**Figure S1. Posterior predictive model checking for the Bayesian normal random‐effects model.** (A) Histograms presenting the observed standardized mean differences (SMDs) in IQ between preterm- and term-born children (leftmost histogram) and the 19 first of 2000 posterior predictive replications. Each replicated dataset consists of 58 independent draws from a normal distribution with mean $\mu$ and variance $\tau^{2}$, where ($\mu, \tau^{2}$) are sampled from the joint posterior distribution. (B) Posterior predictive distributions of extreme values. Left panel: smallest observation of the observed IQ differences (the vertical line at the left of the graph), compared to the smallest observations from each of the 2000 posterior predictive simulated datasets. Right panel: largest observation of the observed IQ differences (the vertical line at the middle of the graph), compared to the largest observations from each of the 2000 posterior predictive simulated datasets. (C) Bayesian standardized residual plot for a single posterior random draw, showing residuals against estimated study-level effects. The horizontal red line at zero indicates perfect model fit.

**Figure S2.** (A) Forest plot of the estimated study-specific effects using the mixture of two normal distributions model with information on prematurity birth level within each study, component assignment for each study and the respective probability (Pr). Red and black vertical lines represent the first and second component means (B) Forest plot of the estimated study-specific effects using the mixture of two t-distributions model with information on prematurity birth level and mean gestational age within each study, component assignment for each study and the respective probability (Pr). Red and black studies are those belonging to the first and second component. Red and black vertical lines represent the first and second component means. EPT, Extremely preterm-born; GA, Gestational Age; IQ, Intelligence Quotient; SMDs, Standardized mean differences; VPT, Very preterm-born**.**

**Figure S3.** Diagnostic plots for **(A)** the mixture of two normal distributions and **(B)** the mixture of two t-distributions models. The joint posterior distribution of the difference between the two component means and the mixture probability is shown. Grey points correspond to random samples from the Marcov Chain Monte Carlo (MCMC) iterations. Blue lines represent posterior density contours at levels: 50%, 75%, and 95%. The top and right marginal histograms display the posterior distributions of the mixture probability and the difference between component means, respectively. The horizontal dashed line indicates the threshold beyond which the two-components model departs from a single-component representation.

**Figure S4.** Forest plot of the estimated study-specific effects using the Dirichlet Process model with information on study characteristics and cluster membership based on co-clustering probabilities (Pr). Studies were further sorted by cluster assignment, prematurity birth level and by decreasing mean gestational age (GA). The colored vertical lines represent the means of the identified main clusters. Black, blue and red studies are those belonging to the first, second and third clusters, respectively, while studies in grey form two to four-studies clusters. na indicates missing values. EPT, Extremely preterm-born; IQ, Intelligence Quotient; SES, socioeconomic factors; SMDs, Standardized mean differences; VPT, Very preterm-born.

**Figure S5.** Posterior distributions of the study-specific weights $p_{i}$ for each study included in the meta-analysis. The posterior distributions are summarized using a forest plot. Blue lines correspond to studies showing no evidence of conflict, while red lines correspond to studies identified as conflicting, defined as having posterior mean weights substantially greater than one.

**Figure S6.** Forest plot presenting the estimated mean Intelligence Quotient (IQ) differences between preterm- and term-born children using only studies published within the last 10 years from the models that reached convergence. Prediction intervals are also showing with orange. DP, Dirichlet Process; EPT, Extremely preterm-born; IQ, Intelligence Quotient; SMDs, Standardized mean differences; VPT, Very preterm-born.

| **Table S1.** Univariable and multivariable meta-regression results. | | | | | | | |
| --- | --- | --- | --- | --- | --- | --- | --- |
|  |  |  | **Coefficients** | |  | **Coefficients** | |
| **Covariates** | **Model** | **Number of studies** | **Median birth year (per 1-year increase)** | **Other covariates** | $\boldsymbol{\tau}^{\mathbf{2}}$ | **P (median birth year)** | **P (other characteristics)** |
| **Median birth year** | linear | 58 | -0.002 [-0.01, 0.01] |  | 0.11 | 0.75 |  |
|  | linear | 15 (EPT subgroup) | -0.008[-0.03,0.01] |  | 0.07 | 0.44 |  |
|  | linear | 43 (VPT subgroup) | -0.001[-0.01,0.01] |  | 0.14 | 0.86 |  |
| **Median birth year** | restricted cubic spline with quantile knots  (1980,1990, 2000,2010) | 58 | -0.05[-0.12,0.01]  0.10[-0.09, 0.28]  -0.18[-0.69, 0.33] |  | 0.11 | 0.12  0.32  0.48 |  |
|  | restricted cubic spline with quantile knots  (1980,1990, 2000,2010) | 15 (EPT subgroup) | -0.01 [-0.13, 0.10]  -0.03[0.34, 0.28]  0.14 [-0.73,1.00] |  | 0.08 | 0.84  0.86  0.76 |  |
|  | restricted cubic spline with quantile knots  (1980,1990, 2000,2010) | 43 (VPT subgroup) | -0.07[-0.16, 0.02]  0.16[-0.10, 0.42]  -0.36[-1.05, 0.34] |  | 0.13 | 0.10  0.22  0.32 |  |
| **Median birth year + mean gestational age** |  | 54 | 0.0001[-0.01, 0.01] | 0.05[-0.02, 0.12] | 0.12 | 0.98 | 0.15 |
| **Mean birth year + type of IQ test** |  | 58 | -0.002[-0.01, 0.01] | 0.08[-0.13, 0.29] | 0.11 | 0.73 | 0.45 |
| **Median birth year + matched/ adjusted for socioeconomic characteristics** |  | 58 | -0.001[-0.01, 0.01] | 0.03[-0.21, 0.27] | 0.12 | 0.86 | 0.78 |
| **Median birth year + study quality assessment score** |  | 58 | -0.003[-0.02, 0.01] | -0.03[-0.10,0.05] | 0.12 | 0.59 | 0.50 |
| **Median birth year + mean gestational age + type of IQ test + matched/ adjusted for socioeconomic characteristics + study quality assessment score** |  | 54 | -0.001[-0.01,0.01] | 0.04[-0.03,0.12]  0.06[-0.16,0.27]  0.06[-0.21,0.33]  -0.03[-0.12,0.05] | 0.12 | 0.87 | 0.26  0.59  0.69  0.45 |
| Abbreviations: EPT, Extremely preterm-born; IQ, Intelligence Quotient; VPT, Very preterm-born. | | | | | | | |

**Table S2.** Prior distributions used for the parameters of the Bayesian models of the main analysis.

| **Models** | **Framework fitted** | **Within-study distribution** | **Between-study distribution** |  | **Prior distributions for key parameters** | | | | | | | | | **DP truncation points (**$\mathbf{N}$**)** |
| --- | --- | --- | --- | --- | --- | --- | --- | --- | --- | --- | --- | --- | --- | --- |
|  |  |  |  | **mean (**$\boldsymbol{\mu}$**) or component mean**  **(**$\boldsymbol{\mu}_{\boldsymbol{i}}$**)** | **prior for the between-study standard deviation**  **(**$\boldsymbol{\tau}$**) or the inverse of the component variance**  **(**${\tau_{i}}^{-2}$**)** | **component** $\mathbf{p}_{\mathbf{i}}$ | **base mean**  **(**$\boldsymbol{\mu}_{\boldsymbol{b}}$**)** | **base variance**  ${(\tau}_{b}\mathbf{)}$ | **concentration (**$\boldsymbol{\alpha}$**)** | **location (**$\boldsymbol{\xi}$**)** | **scale**  **(**$\boldsymbol{\omega}$**)** | **shape (**$\boldsymbol{a}$**)** | **degrees of freedom *(***$\boldsymbol{\nu}$**)**  **or**  **degrees of freedom (**$\boldsymbol{\nu}_{\mathbf{i}}$**)** |  |
| **Normal** | Bayesian | Normal | Normal | $\mu\sim N\left( 0, {10}^{4} \right)$ | $\tau\sim U\left( 0,10 \right)$ |  |  |  |  |  |  |  |  |  |
| **t-distribution** | Bayesian | Normal | t-distribution |  |  |  |  |  |  | $N\left( 0, {10}^{4} \right)$ | $U\left( 0,10 \right)$ |  | $\nu\sim\mathrm{Exp}\left( 0.10 \right)$  $\nu\geq2.5$ |  |
| **Skew-normal** | Bayesian | Normal | Skew-Normal |  |  |  |  |  |  | $N\left( 0, {10}^{4} \right)$ | $U\left( 0,10 \right)$ | $N\left( 0,25 \right)$ | *-* |  |
| **Skew-t** | Bayesian | Normal | Skew-t |  |  |  |  |  |  | $N\left( 0, {10}^{4} \right)$ | $U\left( 0,10 \right)$ | $N\left( 0,25 \right)$ | $\nu\sim\mathrm{Exp}\left( 0.10 \right)$  $\nu\geq4.5$ |  |
| **Mixture of two normal distributions** | Bayesian | Normal | Mixture of two normal distributions | $\mu_{i}\sim N\left( m_{1},{t_{1}}^{2} \right)$  $i=1,2$  *with* $\mu_{1}<\mu_{2}$  $m_{1}\sim N\left( 0, {10}^{4} \right)$  $t_{1}\sim\mathrm{HN}(0,1)$ | ${\tau_{i}}^{-2}\sim\Gamma\left( 3,b \right)$  $b\sim\Gamma\left( 0.03,0.03 \right)$  $i=1,2$ | $p_{i}\sim\mathrm{Dir}(1)$ |  |  |  |  |  |  |  |  |
| **Mixture of two t-distributions** | Bayesian | Normal | Mixture of two t-distributions | $\mu_{i}\sim N\left( m_{1},{t_{1}}^{2} \right)$  $i=1,2$  *with* $\mu_{1}<\mu_{2}$  $m_{1}\sim N\left( 0, {10}^{4} \right)$  $t_{1}\sim\mathrm{HN}(0,1)$ | ${\tau_{i}}^{-2}\sim\Gamma\left( 3,b \right)$  $b\sim\Gamma\left( 0.03,0.03 \right)$  $i=1,2$ | $p_{i}\sim\mathrm{Dir}(1)$ |  |  |  |  |  |  | $\nu_{i}\sim\mathrm{Exp} \left( 0.10 \right)$  $\nu_{i}\geq2.5$ |  |
| **DP** | Bayesian | Normal | DPMp(normal base distribution) |  |  | $p_{i}$*~stick &breaking process* | $\mu_{b}\sim N\left( 0, {10}^{4} \right)$ | $\tau_{b}\sim U\left( 0,10 \right)$ | $U\left( 0.3,5 \right)$ |  |  |  |  | 26 |
| Abbreviations: DP, Dirichlet Process; DPMp, Dirichlet Process Mixture of point masses. | | | | | | | | | | | | | | |

| **Table S3.** Mean and variance of the different random-effects distributions. | | | |
| --- | --- | --- | --- |
| **Models** | **Between-study distribution** | **Parameters of interest** | |
|  |  | Mean of the random-effects distribution  $E\left( Y \right)$ | Variance of the random-effects distribution  $\mathrm{Var}\left( Y \right)$ |
| **Normal** | $N\sim(\mu,\tau^{2})$ | $\mu$ | $\tau^{2}$ |
| **t-distribution** | $t\sim\left( \mu,\omega,\nu\right)$ | $\mu$ | $\frac{\omega^{2}\nu}{(\nu-2)}$ |
| **Skew-normal** | $\mathrm{SN}\left( \xi,\omega,a \right)$ | $\mu=\xi+\omega b\delta$with  $b=\sqrt{\frac{2}{\pi}}$ and $\delta=\frac{a}{\sqrt{1+a^{2}}}$ | $\omega^{2}(1-\left( \delta b \right)^{2})$ with  $b=\sqrt{\frac{2}{\pi}}$ *and* $\delta=\frac{a}{\sqrt{1+a^{2}}}$ |
| **Skew-t** | $\mathrm{ST}(\xi,\omega,\nu,a)$ | $\mu=\xi+\omega b_{v}\delta$ with  $b_{v}=\left( \frac{\nu}{\pi} \right)^{\frac{1}{2}}\frac{\Gamma\left( \frac{\nu-1}{2} \right)}{\Gamma\left( \frac{\nu}{2} \right)},$  $\nu>1$ | $\omega^{2}\frac{\nu}{\nu-2}-{b_{\nu}}^{2}\delta^{2}$ with  $b_{v}={(\frac{\nu}{\pi})}^{1/2}\frac{\Gamma(\frac{\nu-1}{2})}{\Gamma(\frac{\nu}{2})}, \nu>1$ and  $\delta=\frac{a}{\sqrt{1+a^{2}}}$ |
| **Mixture of two normal distributions** | $p_{1}N\left( \mu_{1}, \tau_{1}^{2} \right)+p_{2}N\left( \mu_{2}, \tau_{2}^{2} \right)$ | $\sum_{i=1}^{2} p_{i}\mu_{i}$ | $\sum_{i=1}^{2} p_{i}\tau_{i}^{2}+\sum_{i=1}^{2} {p_{i}(\mu_{i}-E\left( Y \right))}^{2}$ |
| **Mixture of two t-distributions** | $p_{1}t\left( \mu_{1}, \tau_{1}^{2},\nu_{1} \right)+p_{2}t\left( \mu_{2}, \tau_{2}^{2},\nu_{2} \right)$ | $\sum_{i=1}^{2} p_{i}\mu_{i}$ | $\sum_{i=1}^{2} p_{i}\tau_{i}^{2}+\sum_{i=1}^{2} {p_{i}(\mu_{i}-E\left( Y \right))}^{2}$ |
| **DP** | $\mathrm{DPMp}\left( \alpha, F_{o} \right)$ | $\sum_{i=1}^{26} p_{i}z_{i}$*,*  $z_{i}: point masses$  $z_{i}\sim F_{0}$ | $\sum_{i=1}^{26} p_{i}{z_{i}}^{2}-({\sum_{i=1}^{26} p_{i}z_{i})}^{2}$ |
| Abbreviations: DP, Dirichlet Process; DPMp, Dirichlet Process Mixture of point masses | | | |

**Table S4.** Prior distributions and definition of model parameters for all the models used in the sensitivity analyses.

| **Models** | **Framework fitted** | **Within-study distribution** | **Between-study distribution** |  | **Prior distributions for key parameters** | | | | | | | | | **DP truncation points (**$\mathbf{N}$**)** |
| --- | --- | --- | --- | --- | --- | --- | --- | --- | --- | --- | --- | --- | --- | --- |
|  |  |  |  | **mean (**$\boldsymbol{\mu}$**) or component mean**  **(**$\boldsymbol{\mu}_{\mathbf{i}}$**)** | **square root of between-study variance**  **(**$\boldsymbol{\tau}$**)** | **component** $\mathbf{p}_{\mathbf{i}}$ | **base mean**  **(**$\boldsymbol{\mu}_{\mathbf{b}}$**)** | **base variance**  $\boldsymbol{(\tau}_{\mathbf{b}}\mathbf{)}$ | **concentration**  **(**$\boldsymbol{\alpha}$**)** | **Location**  **(**$\boldsymbol{\xi}$**)** | **scale**  **(**$\boldsymbol{\omega}\mathbf{)}$ | **shape**  **(**$\mathbf{a}$**)** | **degrees of freedom (**$\boldsymbol{\nu}_{\mathbf{i}}$**)** |  |
| **Normal(a)** | Frequentist | Normal | Normal |  | REML |  |  |  |  |  |  |  |  |  |
| **t-distribution(a)** | Frequentist | Normal | t-distribution |  | ML |  |  |  |  |  |  |  |  |  |
| **Mixture of two normal distributions with common component mean(a)** | Frequentist | Normal | Mixture of two normal distributions with common mean |  | ML | $p$ = estimated proportion of studies belonging to the component that accounts for extreme effects |  |  |  |  |  |  |  |  |
| **Mixture of two normal distributions with common component mean(b)** | Frequentist | Normal | Mixture of two normal distributions with common mean |  |  | $p=\frac{u^{2}}{u^{2}+u_{out}^{2}}$  $u^{2}=variance of$  $\mathrm{homogeneous}$  $\mathrm{studies}$  $u_{\mathrm{out}}^{2}=variance of$  $\mathrm{outliers}$ |  |  |  |  |  |  |  |  |
| **Mixture of normal and exponentially modified normal distributions** | Frequentist | Normal | Mixture of a normal and an exponentially modified normal distributions |  |  | $p=\frac{u^{2}}{u^{2}+\kappa^{-2}+\lambda^{-2}}$  $u^{2}=variance of$  $\mathrm{homogeneous}$  $\mathrm{studies}$  $\kappa^{-2}+\lambda^{-2}=$  $variances of the$  $\mathrm{exponentially}$  $modified normal$  $\mathrm{distribution}$ |  |  |  |  |  |  |  |  |
| **Normal(b)** | Bayesian | Normal | Normal | $\mu\sim N\left( 0, {0.255}^{2} \right)$ | $\tau\sim U\left( 0,10 \right)$ |  |  |  |  |  |  |  |  |  |
| **t-distribution(b)** | Bayesian | Normal | t-distribution |  |  |  |  |  |  | $N\left( 0, {0.255}^{2} \right)$ | $U\left( 0,10 \right)$ |  | $\nu\sim\mathrm{Exp}\left( 0.10 \right)$  $\nu\geq2.5$ |  |
| **Skew-normal** | Bayesian | Normal | Skew-Normal |  |  |  |  |  |  | $N\left( 0, {0.255}^{2} \right)$ | $U\left( 0,10 \right)$ | $N\left( 0,25 \right)$ |  |  |
| **Skew-t** | Bayesian | Normal | Skew-t |  |  |  |  |  |  | $N\left( 0, {0.255}^{2} \right)$ | $U\left( 0,10 \right)$ | $N\left( 0,25 \right)$ | $\nu\sim\mathrm{Exp}\left( 0.10 \right)$  $\nu\geq4.5$ |  |
| **Mixture of two normal distributions** | Bayesian | Normal | Mixture of two normal distributions | $\mu_{i}\sim N\left( m_{1},{t_{1}}^{2} \right)$  $i=1,2$  *with* $\mu_{1}<\mu_{2}$  $m_{1}\sim N\left( 0, {0.255}^{2} \right)$  $t_{1}\sim\mathrm{HN}(0,1)$ | ${\tau_{i}}^{-2}\sim\Gamma\left( 3,b \right)$  $b\sim\Gamma\left( 0.03,0.03 \right)$  $i=1,2$ | $p_{i}\sim\mathrm{Dir}(1)$ |  |  |  |  |  |  |  |  |
| **Mixture of two t-distributions** | Bayesian | Normal | Mixture of two t-distributions | $\mu_{i}\sim N\left( m_{1},{t_{1}}^{2} \right)$  $i=1,2$  *with* $\mu_{1}<\mu_{2}$  $m_{1}\sim N\left( 0, {0.255}^{2} \right)$  $t_{1}\sim HN(0,1)$ | ${\tau_{i}}^{-2}\sim\Gamma\left( 3,b \right)$  $b\sim\Gamma\left( 0.03,0.03 \right)$  $i=1,2$ | $p_{i}\sim\mathrm{Dir}(1)$ |  |  |  |  |  |  | $\nu_{i}\sim\mathrm{Exp} \left( 0.10 \right)$  $\nu_{i}\geq2.5$ |  |
| **DP(f)** | Bayesian | Normal | DPMp(normal base distribution) |  |  | $p_{i}$~stick &breaking process | $\mu_{b}\sim N\left( 0, {0.255}^{2} \right)$ | $\tau_{b}\sim U\left( 0,10 \right)$ | $U\left( 0.3,5 \right)$ |  |  |  |  | 26 |
| **Mixture of scale normal distributions** | Bayesian | Normal | Mixture of scale normal distributions | $\mu_{i}\sim\mathrm{Logistic}(0,2)$  where study-arms $i=1,2$ and  $\mu_{2}=f(\mu_{2},\mu_{1},\tau_{1},\tau_{2},\rho)$ with $\rho$=correlation | $var_{1}={\tau_{1}}^{2}p_{j}$  $var_{2}=$  $(1-\rho^{2}){\tau_{2}}^{2}p_{j}$  $\tau_{i}\sim U\left( 0,5 \right)$  where study-arms $i=1,2$ and studies  $j=1,\ldots, k$ with  $\rho$=correlation and $p_{j}$=study’s weight | $p_{j}=\frac{\nu}{q_{j}}$  ${q_{j}\sim\chi}_{\nu}^{2}$ *,*  with studies $j=1,\ldots,k$  and $\nu$ = degrees of freedom |  |  |  |  |  |  |  |  |
| **DP(a)** | Bayesian | Normal | DPMp(skew-normal base) |  |  | $p_{i}$~stick &breaking process |  |  | $U\left( 0.3,5 \right)$ | $N\left( 0, {10}^{4} \right)$ | $U\left( 0,10 \right)$ | $N\left( 0,25 \right)$ |  | 26 |
| **DP(b)** | Bayesian | Normal | DPMp(skew-t base) |  |  | $p_{i}$~stick &breaking process |  |  | $U\left( 0.3,5 \right)$ | $N\left( 0, {10}^{4} \right)$ | $U\left( 0,10 \right)$ | $N\left( 0,25 \right)$ | $\nu_{i}\sim\mathrm{Exp} \left( 0.10 \right)$  $\nu_{i}\geq2.5$ | 26 |
| **DP(c)** | Bayesian | Normal | DPMp(normal base) |  |  | $p_{i}$~stick &breaking process | $N\left( 0, {10}^{4} \right)$ | $U\left( 0,10 \right)$ | $U\left( 0.3,10 \right)$ |  |  |  |  | 51 |
| **DP(d)** | Bayesian | Normal | DPMp(normal base) |  |  | $p_{i}$~stick &breaking process | $\mu_{b}\sim N\left( 0, {10}^{4} \right)$ | $U(0, 10)$ | $\Gamma\left( 1,1 \right)$ |  |  |  |  | k |
| **DP(e)** | Bayesian | Normal | DPMp(normal base distribution) |  |  | $p_{i}$~stick &breaking process | $\mu_{b}\sim N\left( 0, {10}^{4} \right)$ | $\tau_{b}\sim U\left( 0,10 \right)$ | $U\left( 0.3,5 \right)$ |  |  |  |  | 61 |
| Abbreviations: DP, Dirichlet Process; DPMp, Dirichlet Process Mixture of point masses; ML, Maximum Likelihood; REML, Restricted Maximum Likelihood. | | | | | | | | | | | | | | |

**Skeptical prior for the pooled standardized mean difference**

As a sensitivity analysis, we imposed a skeptical prior on the model-specific parameters related to the pooled standardized mean difference (SMD). Specifically, the vague normal prior $N\left( 0, {10}^{4} \right)$previously assigned to $\mu, m_{1}, \xi$ and $\mu_{b}$ was replaced with a normal prior centered at zero, reflecting prior skepticism about large effects^1^.

The skeptical prior was specified as

$$N\left( 0, {0.225}^{2} \right)$$

corresponding to a skepticism threshold of $\delta=0.5$, which was considered implausibly large a priori for the pooled effect. Assuming that only 5% of the prior mass lies outside the interval $(-\delta,+\delta)$ that is, $P\left( \left| \mu\right|>\delta\right)=0.05$ which implies a prior standard deviation of SD=$\frac{0.5}{1.96}\approx0.255$. Under this skeptical prior specification, if the magnitude of the effect (i.e. posterior 95% credible intervals and the prediction intervals) and the heterogeneity estimation remain materially unaffected, the data provide strong evidence supporting lower IQ scores among preterm-born children.

**Table S5.** Estimated mean, variance and weight for each component from models using mixtures of two normal and two t-distributions.

| **Model-components** | **Mean with 95%CrI** | **Length of CrI of mean** | **Variance with 95% CrI** | **Length of CrI of variance** | **Weight** |
| --- | --- | --- | --- | --- | --- |
| **Mixture of two normal distributions** | | | | | |
| Component 1 | -1.01[-2.61, -0.81] | 1.80 | 0.14[0.03, 0.53] | 0.50 | 0.36 |
| Component 2 | -0.83[-0.95, -0.50] | 0.45 | 0.10[0.03, 0.39] | 0.36 | 0.64 |
| **Mixture of two t-distributions** | | | | | |
| Component 1 | -0.96[-2.09, -0.81] | 1.28 | 0.13[0.03, 0.48] | 0.45 | 0.47 |
| Component 2 | -0.80[-0.94, -0.35] | 0.60 | 0.11[0.03, 0.46] | 0.43 | 0.53 |
| Abbreviation: CrI, Credible Intervals. | | | | | |

**Table S6.** Summary estimates of the mean Intelligence Quotient (IQ) differences between preterm- and term-born children for all models used in the sensitivity analysis, together with the between-study variance and their corresponding confidence (CI) or credible (CrI) and prediction (PrI) intervals. For Bayesian models, the probabilities that the mean IQ of preterm children is lower than that of term-born children and the probabilities that a new study would find lower IQ scores in preterm compared with term-born children are also presented.

| **Random-effects distribution** | **SMDs with 95% CI/CrI** | **Between-study variance with 95% CI/CrI** | **95% PrI** | $\boldsymbol{Pr}\mathbf{(}\boldsymbol{SMD}\mathbf{<0)}$ | $\boldsymbol{Pr}\mathbf{(}\boldsymbol{SMD}_{\boldsymbol{new}}\mathbf{<0)}$ |
| --- | --- | --- | --- | --- | --- |
| **Normal(a)** | -0.87[-0.98, -0.77] | 0.11[0.07,0.20] | [-1.54, -0.21] |  |  |
| **t-distribution(a)** | -0.87[-0.97, -0.77] | 0.08 |  |  |  |
| **Mixture of normal and exponentially modified normal distributions** | -0.85(se=0.049)  [-0.95, -0.76] | 0.09[0, 0.33] |  |  |  |
| **Mixture of two normal distributions with common component mean(a)** | -0.86[-0.96,-0.76] | 0.09 for homogeneous studies/0.86 considering homogeneous and extreme studies |  |  |  |
| **Mixture of two normal distributions with common component mean(b)** | -0.87(se=0.052)  [ -0.97, -0.77] | 0.06[0.01, 0.37] for homogeneous studies/0.13[1.48, 3.74] additional variance of extreme studies |  |  |  |
| **Normal(b)** | -0.85[-0.95, -0.74] | 0.14[0.09, 0.23] | [-1.56, -0.08] | 1 | 0.98 |
| **t-distribution(b)** | -0.84[-0.92, -0.75] | 0.14[0.09, 0.24] | [-1.44, -0.23] | 1 | 0.98 |
| **Skew-normal** | -0.88[-0.98, -0.79] | 0.15[0.10, 0.23] | [-1.61, -0.35] | 1 | 1 |
| **Skew-t** | -0.88[-0.98, -0.79] | 0.15[0.09, 0.25] | [-1.63, -0.37] | 1 | 1 |
| **Mixture of two normal distributions** | -0.88[-1.00, -0.76] | 0.14[0.08, 0.27] | [-1.66, -0.19] | 1 | 0.99 |
| **Mixture of two t-distributions** | -0.87[-0.98, -0.76] | 0.14[0.08, 0.28] | [-1.57, -0.08] | 1 | 0.98 |
| **DP(f)** | -0.86[-0.99, -0.74] | 0.17[0.09, 0.50] | [-1.44, -0.12] | 1 | 0.98 |
| **Mixture of scale normal distributions** | -0.83 [-0.93, -0.73] | 0.06[0.03,0.11] | [-1.61, -0.07] | 1 | 0.98 |
| **DP(a)** | -0.89[-1.02,-0.79] | 0.14[0.08, 0.34] | [-1.76, -0.23] | 1 | 1 |
| **DP(b)** | -0.89[-1.01,-0.78] | 0.14[0.08, 0.34] | [-1.81, -0.25] | 1 | 1 |
| **DP(c)** | -0.89[-1.01, -0.78] | 0.14[0.08,0.29] | [-1.61, -0.20] | 1 | 0.99 |
| **DP(d)** | -0.89[-1.01, -0.78] | 0.14[0.08,0.33] | [-1.66, -0.24] | 1 | 1 |
| **DP(e)** | -0.86[-0.98, -0.74] | 0.16[0.08,0.47] | [-1.55, -0.18] | 1 | 0.99 |
| Abbreviations: DP, Dirichlet Process; SMDs, Standardized mean differences. | | | | | |

**Figure S1. Posterior predictive model checking for the Bayesian normal random‐effects model.** (A) Histograms presenting the observed standardized mean differences (SMDs) in IQ between preterm- and term-born children (leftmost histogram) and the 19 first of 2000 posterior predictive replications. Each replicated dataset consists of 58 independent draws from a normal distribution with mean $\mu$ and variance $\tau^{2}$, where ($\mu, \tau^{2}$) are sampled from the joint posterior distribution. (B) Posterior predictive distributions of extreme values. Left panel: smallest observation of the observed IQ differences (the vertical line at the left of the graph), compared to the smallest observations from each of the 2000 posterior predictive simulated datasets. Right panel: largest observation of the observed IQ differences (the vertical line at the middle of the graph), compared to the largest observations from each of the 2000 posterior predictive simulated datasets. (C) Bayesian standardized residual plot for a single posterior random draw, showing residuals against estimated study-level effects. The horizontal red line at zero indicates perfect model fit. Rep, Replication.

In Figure S1, panels A and B indicate noticeable discrepancies between the observed and replicated data under the Bayesian normal random-effects model, particularly in the tails of the distribution. In panel B, the observed minimum IQ difference is more extreme than is typically generated under the posterior predictive distribution, suggesting the presence of studies with extreme left-sided effects more often than expected under a single normal distribution. Additionally, the posterior predictive p-value was 0.98, indicating that replicated data were rarely more extreme than the observed data and raising concerns about the adequacy of the normal random-effects assumption^2^. Panel C further reveals substantial residual variability across the range of estimated study-specific effects, suggesting heterogeneity patterns not fully captured by the normality assumption. Together, these findings motivate the consideration of more flexible random-effects distributions, such as asymmetric or parsimonious mixture models, to better accommodate extreme and heterogeneous study effects.

**Figure S2.** (A) Forest plot of the estimated study-specific effects using the mixture of two normal distributions model with information on prematurity birth level within each study, component assignment for each study and the respective probability (Pr). Red and black vertical lines represent the first and second component means (B) Forest plot of the estimated study-specific effects using the mixture of two t-distributions model with information on prematurity birth level and mean gestational age within each study, component assignment for each study and the respective probability (Pr). Red and black studies are those belonging to the first and second component. Red and black vertical lines represent the first and second component means. EPT, Extremely preterm-born; GA, Gestational Age; IQ, Intelligence Quotient; SMDs, Standardized mean differences; VPT, Very preterm-born**.**

**Figure S3.** Diagnostic plots for **(A)** the mixture of two normal distributions and **(B)** the mixture of two t-distributions models. The joint posterior distribution of the difference between the two component means and the mixture probability is shown. Grey points correspond to random samples from the Marcov Chain Monte Carlo (MCMC) iterations. Blue lines represent posterior density contours at levels: 50%, 75%, and 95%. The top and right marginal histograms display the posterior distributions of the mixture probability and the difference between component means, respectively. The horizontal dashed line indicates the threshold beyond which the two-components model departs from a single-component representation.

Figure S3 presents a diagnostic tool to assess the relationship between the difference in the two component means of the mixture models and the mixture probability. An additional diagnostic quantity reflected in this figure is the posterior probability of having a unique component in the model. For instance, in case of a mixture of two normal distributions with means $\mu_{1}$ and $\mu_{2}$ and variances $\tau_{1}^{2}$and $\tau_{2}^{2}$, the sufficient condition for the mixture to include only one component is given by |$\mu_{1}$*-* $\mu_{1}$*|< 2*$min(\tau_{1},\tau_{2})$ as described by McLachlan, G., & Peel, D.$ADDIN ZOTERO\_ITEM CSL\_CITATION \{"citationID":"FsXOU6Sm","properties":\{"formattedCitation":"\backslash\backslash super 3\backslash\backslash nosupersub\{\}","plainCitation":"3","noteIndex":0\},"citationItems":[\{"id":321,"uris":["http://zotero.org/users/local/XpQwLZeb/items/4XVFG8MU"],"itemData":\{"id":321,"type":"chapter","event-place":"Hoboken","page":"Chap. 6, pp.175-220","publisher":"NJ: Wiley","publisher-place":"Hoboken","title":"Finite Mixture Models","author":[\{"literal":"McLachlan, G., \& Peel, D"\}],"issued":\{"date-parts":[["2000"]]\}\}\}],"schema":"https://github.com/citation-style-language/schema/raw/master/csl-citation.json"\}$^3^ This condition provides a practical threshold for evaluating whether the fitted mixture distribution departs from a single-component representation. In Figure S3, this threshold is indicated by the horizontal dashed line and is evaluated across posterior draws, allowing assessment of whether the data support more than a single component.

In both models, most of the posterior mass of the difference between the two component means concentrates above the separation threshold. In the mixture of two normal distributions model (Figure S3, panel A), the difference between the component means substantially increases only when the mixture probability approaches one suggesting weak identification of the second component and possible influence of a small number of extreme studies, making the necessity of a second component ambiguous. In the mixture of two t-distributions model (Figure S3, panel B), the separation between the two component means remains relatively stable across the range of all plausible mixture probabilities, introducing uncertainty about whether the two components are meaningfully distinct. Overall, these diagnostics suggest **weak to moderate evidence** for a second component, with limited support for a substantively distinct mixture structure.

**Figure S4.** Forest plot of the estimated study-specific effects using the Dirichlet Process model with information on study characteristics and cluster membership based on co-clustering probabilities (Pr). Studies were further sorted by cluster assignment, prematurity birth level and by decreasing mean gestational age (GA). The colored vertical lines represent the means of the identified main clusters. Black, blue and red studies are those belonging to the first, second and third clusters, respectively, while studies in grey form two to four-studies clusters. na indicates missing values. EPT, Extremely preterm-born; IQ, Intelligence Quotient; SES, socioeconomic factors; SMDs, Standardized mean differences; VPT, Very preterm-born.

**Figure S5.** Posterior distributions of the study-specific scale weights $p_{i}$. Studies with large posterior weights $p_{i}$ correspond to conflicting studies. The posterior distributions are summarized using a forest plot. Blue lines correspond to studies showing no evidence of conflict, while red lines correspond to studies identified as conflicting, defined as having posterior median weights substantially greater than one.

**Figure S6.** Forest plot presenting the estimated mean Intelligence Quotient (IQ) differences between preterm- and term-born children using only studies published within the last 10 years and the models that reached convergence. Prediction intervals are denoted with orange. DP, Dirichlet Process; EPT, Extremely preterm-born; IQ, Intelligence Quotient; SMDs, Standardized mean differences; VPT, Very preterm-born.

**JAGS code specification for the standardized effects**

Effect sizes were modeled as standardized mean differences (SMDs) within the hierarchical Bayesian framework. For each study $i=1,\ldots,k$, the group-specific observed mean IQ values ($y_{1,i},y_{2,i}$) were modeled directly using normal likelihoods, with variances ($\sigma_{1,i}^{2},\sigma_{2,i}^{2}$) determined by the reported sample sizes ($n_{1,i},n_{2,i}$) and standard deviations ($sd_{1,i}, sd_{2,i}$) as follows:

For each study $i=1,\ldots,k$ and each arm:

$$y_{1,i}\sim N(mu_{1,i}, \sigma_{1,i}^{2})$$

$$y_{2,i}\sim N(mu_{2,i}, \sigma_{2,i}^{2})$$

$$\sigma_{1,i}^{2}=\frac{\mathrm{sd}_{1,i}^{2}}{n_{1,i}}$$

$$\sigma_{2,i}^{2}=\frac{\mathrm{sd}_{2,i}^{2}}{n_{2,i}}$$

A study-specific baseline mean $mu_{1,i}$ represented the mean IQ in the reference (term-born) group, while the mean IQ in the preterm-born group was expressed as $mu_{1,i} +\delta_{i}*SD_{i,pooled}$, where $\delta_{i}$ denotes the standardized random effects and $SD_{i,pooled}$ is the pooled within-study standard deviation:

$$mu_{1,i}\sim N(0,{10}^{4})$$

$$mu_{2,i}=mu_{1,i}+\delta_{i}*SD_{i,pooled}$$

The pooled standard deviation was calculated using the conventional unbiased estimator based on group-specific variances and sample sizes as follows:

$$SD_{poooled}=\sqrt{\frac{{(n}_{1,i}-1){sd}_{1,i}^{2}+{(n}_{2,i}-1){sd}_{2,i}^{2}}{n_{1,i}+n_{2,i}-2}}$$

Then the standardized random effects $\delta_{i}$ were assumed to arise from a normal or an alternative random-effects distribution.

References:

1.	Spiegelhalter DJ, Abrams KR, Myles JP. *Bayesian Approaches to Clinical Trials and Health‐Care Evaluation*. John Wiley & Sons; 2003.

2.	Gelman A, Carlin JB, Stern HS,et al. *Bayesian Data Analysis*. 3rd ed. 2013. https://doi.org/10.1201/b16018

3.	McLachlan, G., & Peel, D. Finite Mixture Models. NJ: Wiley; 2000.
